# Low-Cost Synthesis of Silicon Quantum Dots with Near-Unity Internal Quantum Efficiency

**DOI:** 10.1021/acs.jpclett.1c02187

**Published:** 2021-09-09

**Authors:** Jingjian Zhou, Jing Huang, Huai Chen, Archana Samanta, Jan Linnros, Zhenyu Yang, Ilya Sychugov

**Affiliations:** † Department of Applied Physics, KTH - Royal Institute of Technology, Stockholm 10691, Sweden; ‡ MOE Laboratory of Bioinorganic and Synthetic Chemistry, Lehn Institute of Functional Materials, School of Chemistry, Sun Yat-sen University, Guangzhou 510275, Guangdong China; § Dongguan Institute, Sun Yat-sen University, Dongguan, 523808, China

## Abstract

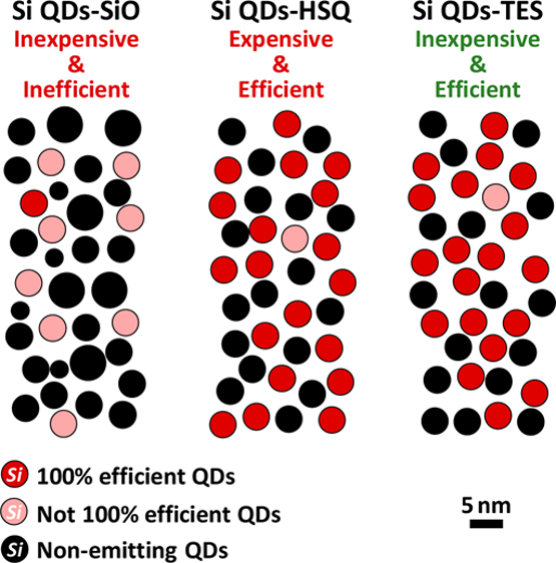

As a cost-effective batch synthesis method, Si quantum dots (QDs) with near-infrared photoluminescence, high quantum yield (>50% in polymer nanocomposite), and near-unity internal quantum efficiency were fabricated from an inexpensive commercial precursor (triethoxysilane, TES), using optimized annealing and etching processes. The optical properties of such QDs are similar to those prepared from state-of-the-art precursors (hydrogen silsesquioxane, HSQ) yet featuring an order of magnitude lower cost. To understand the effect of synthesis parameters on QD optical properties, we conducted a thorough comparison study between common solid precursors: TES, HSQ, and silicon monoxide (SiO), including chemical, structural, and optical characterizations. We found that the structural nonuniformity and abundance of oxide inherent to SiO limited the resultant QD performance, while for TES-derived QDs this drawback can be avoided. The presented low-cost synthetic approach would significantly favor applications requiring high loading of good-quality Si QDs, such as light conversion for photovoltaics.

Owing to natural element abundance, nontoxicity, and unique optoelectronic properties, silicon quantum dots (Si QDs) have emerged as an attractive class of QDs, especially in the applications of biolabeling,^1−3^ quantum-dot-based light-emitting diodes (QLEDs),^4,5^ and semitransparent photovoltaics (PVs).^6,7^ Benefiting from wavelength-tunable photoluminescence (PL),^8−10^ high photoluminescence quantum yield (PLQY),^11^ and a significant Stokes shift,^12^ Si QDs are considered as promising fluorophores in LEDs^13,14^ and luminescent solar concentrators (LSCs).^15,16^ For example, Si QD-based LEDs have achieved a record external quantum efficiency (EQE) of 6.2%,^4^ and an optical power efficiency of 7.9% was obtained for an LSC prototype (9 × 9 cm^2^) based on Si QDs/polymer nanocomposites.^6^ Nevertheless, large quantities of Si QDs with a high PLQY of core-related luminescence are essential for the practical application of these technologies. Therefore, a low-cost synthesis approach of Si QDs with good quality is highly demanded.

Various methods have been developed for the synthesis of colloidal Si QDs, including top-down approaches, such as electrochemical Si wafer etching^17,18^ and laser ablation of solid Si;^19^ bottom-up approaches, such as reduction reaction of halosilanes;^20^ and precursor decomposition and reassembly approaches, such as plasma synthesis from silane gas^21,22^ and thermal pyrolysis of silicon-rich oxide compounds.^23−27^ However, very few methods are suitable for the scalable production of Si QDs with a decent PLQY. One of them is a thermal pyrolysis of silicon oxide materials with a Si excess, such as hydrogen silsesquioxane (HSQ).^27^ This is a highly pure material with a well-defined cage structure, which translates, however, to a costly synthesis. This HSQ-based method is widely used to prepare ligand-passivated Si QDs with emission spanning from the visible to the near-infrared regime (700–1050 nm)^10^ and a high PLQY (up to 60%, depending on the emission wavelength).^28,29^ With this approach, a hybrid nanocomposite (up to ∼65% PLQY at 800 nm) comprising Si QDs and an off-stoichiometric thiol-ene (OSTE) polymer host matrix was reported for applications requiring high-quality Si QDs in a solid phase.^11^ However, the high cost of HSQ hinders the mass production of Si QDs in practice.

Other starting materials with a less-defined structure and composition, hence a lower cost, have also been investigated. For example, the preparation of Si QDs using thermal-induced disproportionation of silicon monoxide (SiO) was developed.^30,31^ Unfortunately, the PLQY of the Si QD ensemble (PL peak at 835 nm) was relatively low (≤15%), insufficient for light-conversion applications (at least PLQY ≥ 50% is typically required). Recently, a sol–gel polymerization reaction from inexpensive starting materials, such as HSiCl_3_, trimethoxysilane (TMS), and triethoxysilane (TES), has emerged as another low-cost alternative to the expensive HSQ.^25,32,33^ Here, unlike for HSQ, the resulting HSiO_1.5_ polymers contain impurities, such as carbon. For instance, colloidally stable, alkyl-capped Si QDs (PLQY ≤ 25% at 955 nm peak emission) were obtained from the reductive thermolysis of (HSiO_1.5_)*_n_* sol–gel glasses.^2,34^ Saitow et al. reported a scalable and cost-effective method starting from HSiCl_3_ relying on thermal treatment of xerogels from TMS. The TMS-derived HSQ polymer, indeed, is an inexpensive source with 380 times lower cost than HSQ. However, the PLQY of TMS-derived Si QDs was limited up to 25% (PL peak position at 800 nm), and the mass yield difference of the final product was not accounted for.^33^ Shirahata et al. applied ligand-passivated Si QDs synthesized from TES (PLQY ≤ 30%) to white LEDs^13^ and deep issue imaging.^35^ However, this recipe highly relies on size-selected purifications, which is unrealistic for mass production. Although extensive efforts were invested in this aspect,^25,36−39^ still no emission with a high PLQY from such Si QDs has been reported. Consequently, as of now, there is no real cost-effective synthesis method available for large-scale production of good-quality Si QDs with core-related luminescence. This limitation will significantly obstruct the development of promising technologies based on Si QDs.

Here, we present an optimized synthesis for Si QDs, based on a sol–gel polymer starting from an inexpensive TES. As a cost-effective synthesis approach, Si QDs with a near-infrared PL (∼850 nm peak), a high PLQY of ∼55% in OSTE (∼40% in toluene), and near-unity internal quantum efficiency (IQE) were fabricated (referred to as QDs-TES). We report our findings as a comparison study between HSQ (QDs-HSQ)- and SiO (QDs-SiO)-derived nanoparticles with a similar PL peak position and an identical ligand passivation. Thorough structural, chemical, and optical characterizations were performed to understand the similarities and differences between these nanomaterials. Results indicate that the intrinsic nonuniformity and the oxide phase excess prevent fabrication of good-quality QDs from SiO. TES-derived QDs, however, can be controlled sufficiently to replace HSQ as a standard starting material for Si QD synthesis.

In Figure 1, the molecular formula and structures of three silicon-rich precursors used here, HSQ, SiO, and TES, are illustrated. Corresponding annealing conditions are also provided (see detailed descriptions of optimized experimental conditions in section S1, Supporting Information). As the most commonly used precursor for the batch synthesis of Si QDs, HSQ has been comprehensively studied.^27^ The constitutional unit of this well-defined molecular precursor, [HSiO_1.5_]_*n*_ (*n* = 8), is a cage-like structure with Si–O–Si frameworks and tetrahedral Si vertices. During the thermal process, the pyrolysis of Si–H groups of HSQ generates silane molecules, which diffuse throughout the surrounding matrix and decompose to form Si clusters.^26^ Through crystallization at a high temperature, Si QDs are formed in an SiO_2_ matrix. As for TES molecules, specific pretreatments, including hydrolysis and condensation, are required to transform them into sol–gel polymer and then dried them into xerogels for subsequent thermal annealing. The TES-derived xerogel, which is carbon-containing (unlike HSQ), is another kind of silsesquioxane with a formula [RSiO_1.5_]_*n*_ (R is either an ethoxy group or a hydrogen atom). The structure of these TES-derived xerogels is a mixture of cages and cross-linked networks.^33,40^ The formation of Si QDs during annealing is similar to HSQ, except that the ethoxy groups in the TES-derived xerogels can reduce the energetic barrier for diffusion of Si atoms throughout the oxide network at the stage of Si crystallization.^25^ As a result, compared to HSQ, a lower annealing temperature is required for these TES-derived xerogels, as shown in Figure 1. However, the presence of carbon may lead to undesirable impurities in the final material. As for SiO, it consists of silicon clusters and an amorphous SiO_*n*_ (1 < *n* < 2) matrix.^41^ The intrinsic instability of the monoxide phase prone to complete oxidation and inherent material nonuniformity make it difficult to define its structure succinctly.

**Figure 1:**
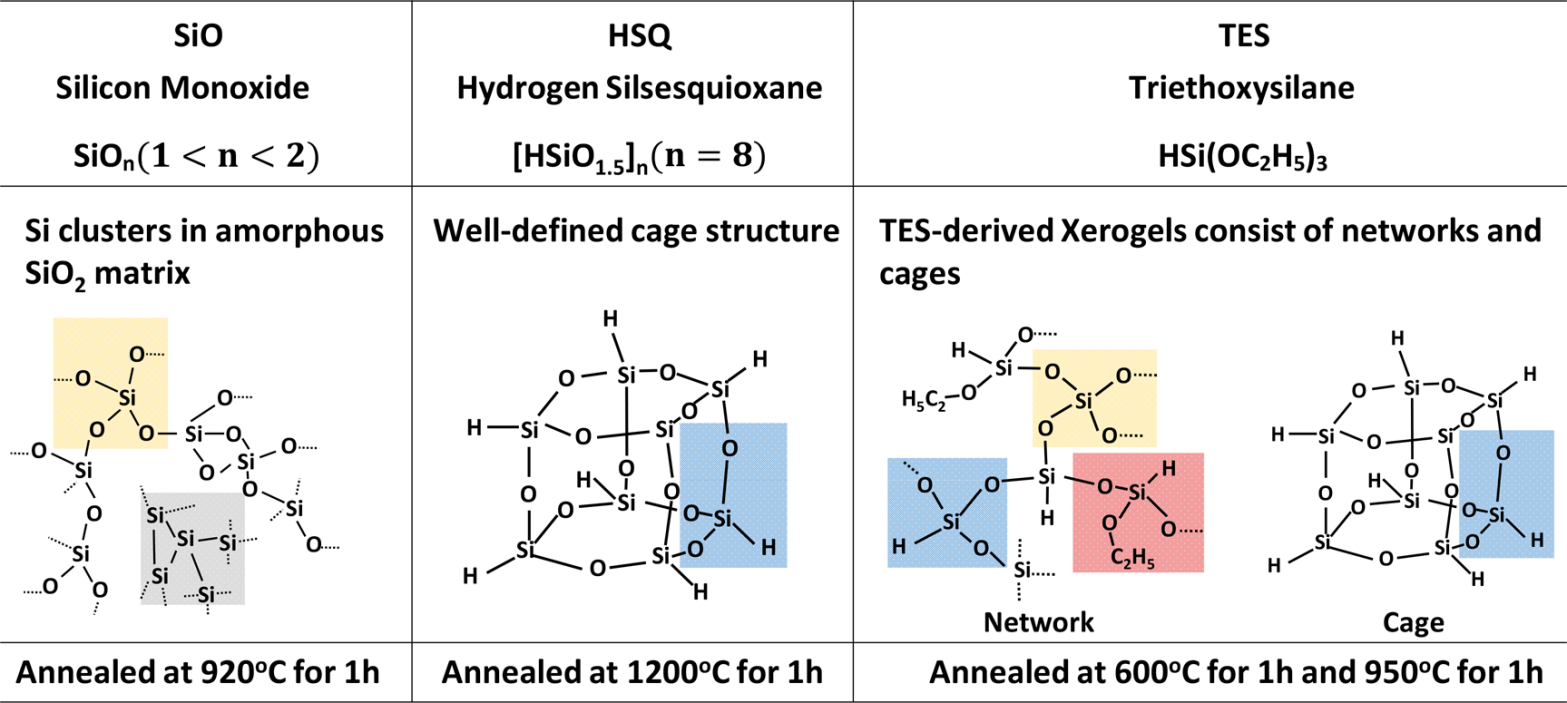
Three types of silicious precursors for the synthesis of Si QDs, their chemical formulas, and illustrations of their molecular structures. For TES, there are a series of pretreatments to form xerogels for the subsequent annealing.

In all these samples, the high-temperature annealing leads to the formation of a nanocrystalline silicon phase. To demonstrate this fact, we carried out XRD measurements of three preannealed powders as a reference (Supporting Informaton, section S2, Figure S1) and the annealed HSQ (blue), TES-derived xerogels (green), and SiO (red) powders (Figure 2a). The broad signals centered at 21° in the reference XRD patterns are attributed to amorphous silicon dioxide.^42^ For preannealed SiO powders, the additional humps centered at 28°, 50° are ascribed to the broadenings of the (111) (220) (311) diffraction of Si nanoclusters.^43^ The characteristic peaks of Si nanocrystals located at 28°, 48°, and 55° are evident in all three annealed powders, whereas they are absent from all reference samples. This confirms that Si QDs are formed through thermal annealing. It is important to note that the intensity difference in the crystalline Si signals is attributed to the various mass percentages of Si QDs in the annealed samples, which will be further discussed later.

**Figure 2:**
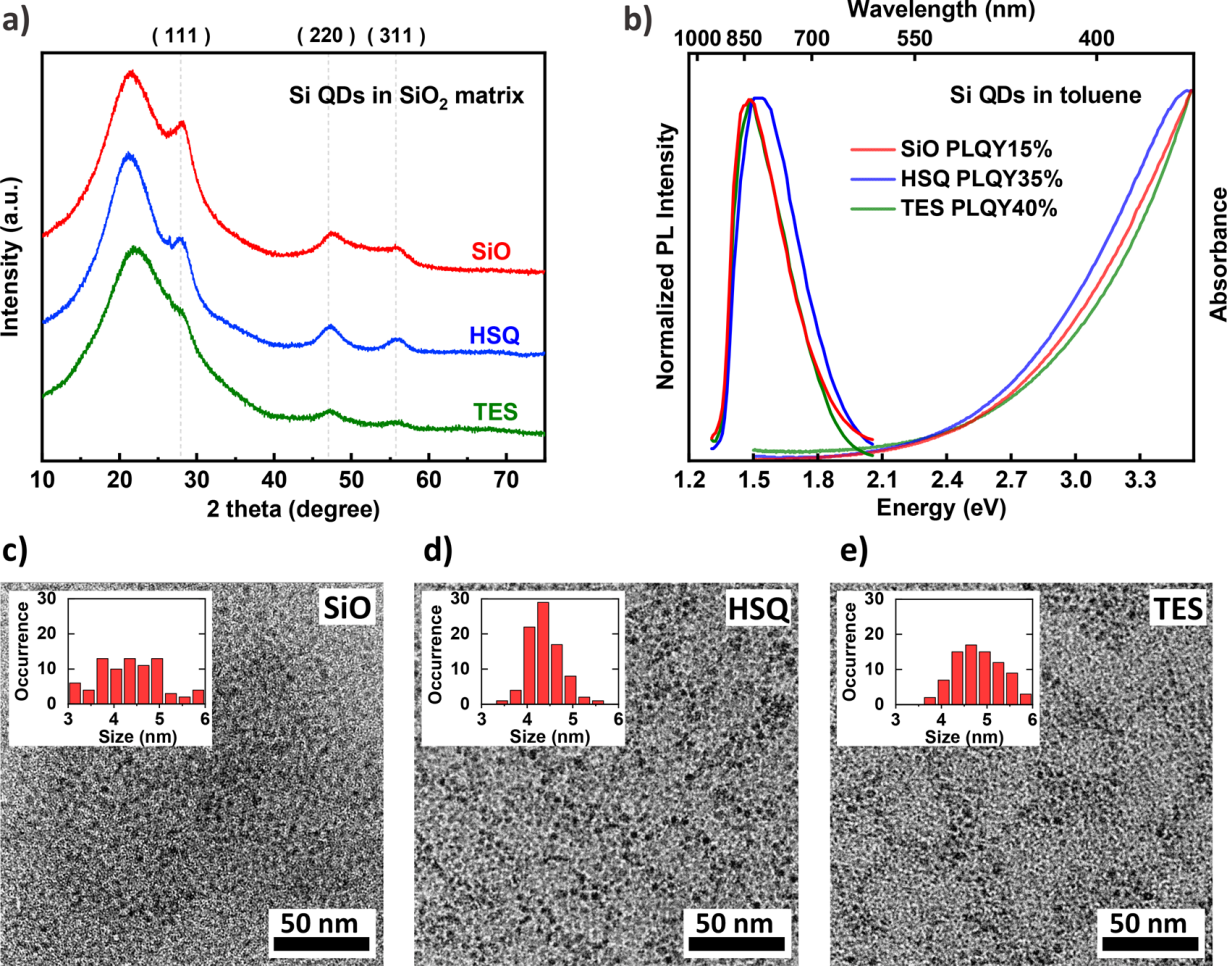
Chemical, optical, and structural characterizations of Si QDs synthesized from three precursors. (a) X-ray powder diffraction (XRD) spectra of the annealed HSQ (1200 °C), annealed TES-derived xerogels (950 °C), annealed SiO (920 °C). (b) Emission and absorption spectra of Si QDs in toluene synthesized from three precursors. (c–e) Transmission electron microscope (TEM) images of these Si QDs. The inset of each TEM image is the size distribution of QDs.

The absorption and PL emission spectra of Si QDs from three precursors (Figure 2b) reveal that they have almost the same absorption edge onset and PL emission peak position. In general, two distinct PL bands, the “S-band” and the “F-band”, are known for Si QDs with entirely different mechanisms of emission.^44^ The “S-band”, with yellow to near-infrared PL and a slow microsecond lifetime, corresponds to the core-related PL from the quasidirect-bandgap transitions of Si QDs and can be tuned by the quantum confinement effect. In contrast, the “F-band”, with a blue to yellow range and a fast nanosecond PL lifetime, likely originates from the surface carbon-based groups or chromophores. Here, we focus on QDs solely with the “S-band”, having a significant Stokes shift, which is suitable for light-converting applications due to low reabsorption. Although the emission and absorption characteristics of all three types of Si QDs studied here are identical, their corresponding PLQY values differ. With a similar size of the QDs and the same ligand (methyl 10-undercenoate) for surface passivation, the PLQY of the ensemble QDs-TES in toluene is ∼40%, similar to that of QDs-HSQ (∼35%). However, the PLQY of the ensemble QDs-SiO is only ∼15% even after recipe optimization (Supporting Information, section S3, Table S1), close to previous record values.^45^

According to previous reports,^10,46^ the ∼850 nm center wavelength corresponds to a Si QD size of 4–5 nm. This is consistent with the size of QDs measured from TEM, as shown in Figure 2c–e. The inset of each TEM image depicts the size distribution of QDs. Indeed, the mean size of Si QDs synthesized from all three precursors was 4–5 nm. However, QDs-SiO were not as uniform as the other two. From another low-magnification TEM image of the QDs-SiO sample (Figure S2), larger agglomerates are evident. The nonuniformity of QDs-SiO possibly reflects the nonuniformity of SiO, where widely distributed Si clusters serve as crystallization centers during thermal annealing. In contrast, the size distributions of QDs-HSQ and QDs-TES are nearly monodispersed.

Although TES has been used as the precursor for Si QDs synthesis previously, the resulting Si QDs always exhibited emission with a low PLQY, unsuitable for mass production of high-quality Si QDs. In this work, we successfully modified the synthesis procedures, mainly at the annealing and etching steps, and the PLQY of as-synthesized colloidal QDs was successfully enhanced.

First, the effect of annealing temperature on the peak position of the PL emission of QDs-TES was carefully investigated. The TGA curve of the TES-derived xerogels (Figure S3, green) revealed that from 220 to 450 °C the incomplete hydrolysis (residual TES) and incomplete condensation (residual silanol) product were eliminated at this stage.^47^ Therefore, the TES-derived xerogels were first preannealed at 600 °C (*T*_1_) for 1 h (*t*_1_) to eliminate residual carbon and byproducts in the polymer, as illustrated by the inset in Figure 3, upgrading the main product purity for the subsequent high-temperature annealing. ATR results (Figure S4) show that the aging and drying process can effectively remove excess ethanol and water in the TES xerogels, while annealing at 600 °C for 1 h possibly contributes to further eliminating the byproducts from the incomplete hydrolysis and condensation that were trapped inside the polymer network, as indicated by Figure S3 and Table S2. As indicated in Figure 3, the PL peak position moved to a longer wavelength with a higher annealing temperature at the second step (*T*_2_). Generally, higher temperatures favor the formation of larger particles, which results in red-shifted emission according to the quantum confinement effect.

**Figure 3:**
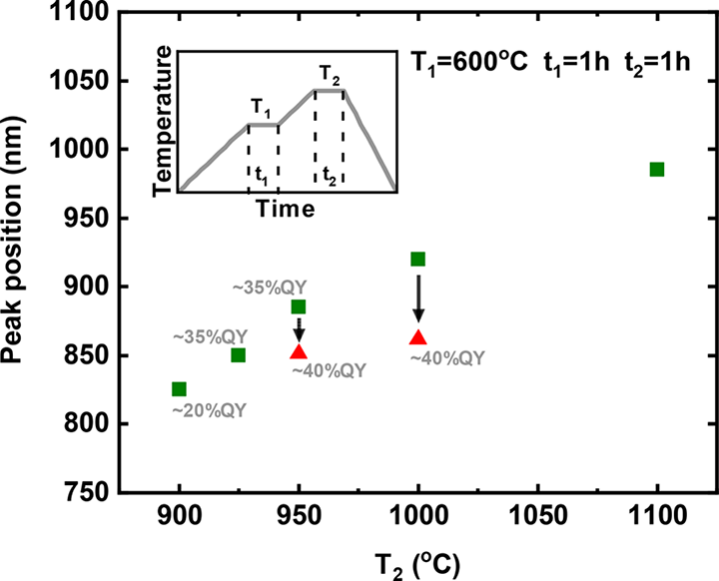
Effect of high annealing temperature on the peak position of the PL emission of Si QDs synthesized from TES (green squares). After HF etching was extended, the peak position can be tuned to the range of ∼850 nm (red triangles). The inset illustrates the two-stage annealing process of TES-derived xerogels. Note that for Si QDs with a peak wavelength exceeding 900 nm, the PLQY values were not shown due to the limitation of detector sensitivity.

Next, HF etching was also recognized as an important step for the final size of QDs-TES. On the one hand, an efficient HF etching process can etch off all the SiO_2_ matrix and liberate all the Si QDs. On the other hand, excess HF (effect of amount of HF used shown in Table S3) can continuously consume surface atoms of Si QDs and make the size of QDs smaller. This is because the etching process was performed in an ambient environment, and it is inevitable that surface atoms of Si QDs would be slowly oxidized by the oxygen in the system and then be removed by the reaction with the excess HF (effect of etching time on PL peak position shown in Table S4). Therefore, to access the as-annealed size of Si QDs (shown as green squares in Figure 3), mild etching conditions (detailed in Table S5) were chosen to liberate free-standing Si QDs from the oxide matrix, minimizing the impact of HF etching on the dimension of Si QD. With extended HF etching (detailed in Table S6), the PL peak position of Si QDs annealed from a higher temperature can be also tailored to the target emission wavelength (shown as red triangles in Figure 3), although the mass yield of Si QDs becomes slightly lower inevitably (the effect of the etching time on the QD mass yield is explicitly shown in Table S7). By comparing the ensemble PLQY of Si QDs in toluene solutions, the optimal annealing condition is 950 °C annealing for 1 h with 1 h HF etching.

We attribute the benefits behind this optimal condition to a good balance of the annealing and etching processes. Under an elevated annealing temperature, the crystallinity of silicon increased, representing a well-ordered, defect-free crystalline silicon core. The extended etching process tailored the size of QDs to the target and simultaneously provided sufficient elimination of oxide around Si QDs, resulting in a PLQY increase. For example, from the third green square (center wavelength ∼885 nm) to the first red triangle (center wavelength ∼850 nm) in Figure 3, it is most likely that both the size modification and the sufficient elimination of surface oxide contribute to the increase of PLQY. A “volcano-shaped” behavior of the size-dependence PLQY of Si QDs was reported in previous studies, revealing that the optimum is located at 820–830 nm.^45^ The extended etching has tuned the PL center wavelength closer to the suggested optimum. The effect of the oxide amount at the QD surface on the PLQY is discussed later. As a result, this optimized recipe can be considered as a good balance of annealing and etching with an acceptable loss of mass yield of Si QDs. When annealed at 1000 °C with subsequent etching for 2 h, the PLQY of Si QDs would not further increase, and the mass yield of Si QDs becomes unacceptably low (shown in Table S7). As for the annealing time at *T*_2_ (*t*_2_), Table S8 indicates that the extended *t*_2_ would favor formation of larger particles but not as effective as elevating the annealing temperature.

Similar optimization was attempted for the synthesis of QDs-SiO (shown in Table S1). However, the PLQY of QDs-SiO in toluene never exceeded 15%. Synthesis of QDs-HSQ followed an already optimized procedure.^6^

Next, solid-phase nanocomposites were fabricated, which is more relevant for applications of Si QDs in devices, such as LSCs. Si QDs were encapsulated in an off-stoichiometric thiol-ene (OSTE) polymer host matrix (experimental details in Supporting Information), which is recognized as an efficient host matrix for Si QDs.^11,48^ After being embedded in OSTE, all samples showed an improved PLQY, enhanced by approximately 10–15 additional percent. The resulting PLQY of ∼55% for QDs-TES in OSTE is comparable with state-of-the-art QDs-HSQ.

Table 1 shows a comparison of the cost of precursors, the QD mass yield, the relative price of QDs, and their efficiencies. With a similar high quality of QDs: ∼50% PLQY in OSTE (∼40% PLQY in toluene), the cost of QDs-TES is approximately 10 times lower than that of QDs-HSQ. This is an important practical result of this work. For example, a luminescent solar concentrator (30 × 30 cm^2^) with ∼100 mg loading of Si QDs would require ∼330 USD using HSQ as the precursor, while only ∼35 USD for commercial TES as the precursor following the recipe introduced here. With regard to QDs-SiO, they are not optically efficient enough for such large-scale practical applications, even though being of very low cost. Indeed, the power conversion efficiency of an LSC is proportional to the PLQY of QDs for small area devices and is even more sensitive to this parameter for large area ones.^49^

**Table 1:** Price of Commercial Precursors, QD Mass Yield from Precursors, the Resulting Relative Price of Si QDs and Their Efficiencies

precursor	price USD/g	QD mass yield from precursor^a^	QD relative price^b^	PLQY
SiO	3.5–7	∼6%	0.15–0.3	25 ± 3% in OSTE
				15 ± 2% in toluene
HSQ	300–360	∼10%	7–15	50 ± 5% in OSTE
				35 ± 4% in toluene
TES	2.5–5	∼1%	1	55 ± 6% in OSTE
				40 ± 4% in toluene

a Note that to estimate mass yields, the mass of Si QDs was evaluated from optical properties (absorption, QY, and emission).

b The relative price of Si QD was calculated based on precursor quotes from different vendors, measured mass losses from annealing, and estimated mass yield of QDs. The price of “QD-TES” was set as “1”. An example of mass yield estimation is given in section S4, Supporting Information.

Finally, the reproducibility of the QD-TES recipe was verified by several batches of synthesis (Supporting Information, section S5, Table S9). The stability of the PLQY of QDs-TES/OSTE nanocomposite over months-long storage in ambient was also confirmed (Figure S5), which is in line with previous reports for OSTE as a stable matrix for QDs.^11,48^ The dependency of the PLQY of QDs-TES/OSTE nanocomposite on the excitation wavelength is presented in Figure S6, showing a uniform response from 400 to 520 nm.

To better understand the differences and similarities in the photophysical properties of these Si QDs, we first performed spectrally resolved PL decay characterization (experimental details in Supporting Information, section S1). An example measured at 878 nm is shown in Figure 4, exhibiting a lifetime of ∼250 μs and a dispersion factor “beta” of 0.92 of the stretched exponential fit. A long microsecond lifetime is a typical sign of core-related PL from Si QDs, corresponding to quantum-confined radiative transitions.

**Figure 4:**
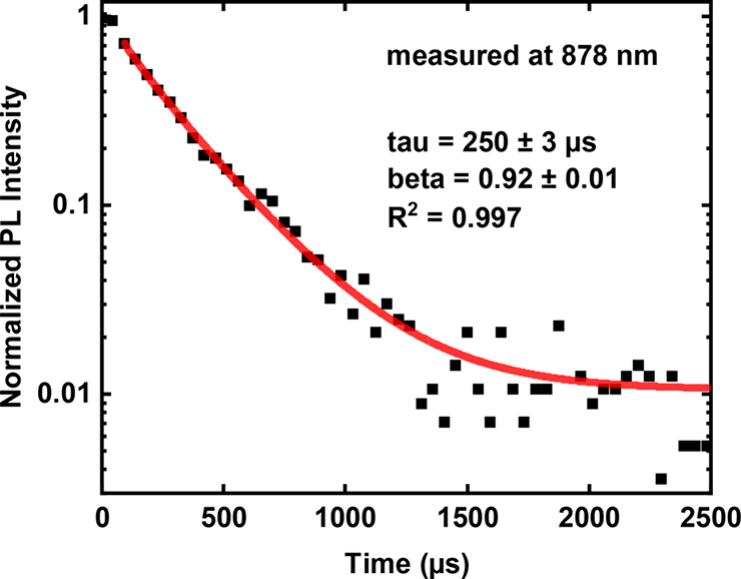
A spectrally resolved PL decay of QDs-TES detected at 878 nm and stretched exponential fit. The decays of QDs-SiO and QDs-HSQ detected at the same wavelength are shown in Figure S7.

A set of measurements was taken at different detection wavelengths to obtain size-dependent recombination rates. Spectrally resolved decays exclude inhomogeneous broadening in an ensemble by detecting only a narrow spectral interval (∼6 nm here). However, because Si QDs have a broad homogeneous line width (∼60 nm at room temperature^50^), a separate procedure is required to exclude this contribution and obtain an intrinsic recombination rate. We therefore used a “numerical size-selection” method developed here^51^ and later adopted by others.^52,53^ After this deconvolution procedure, we obtained nearly monoexponential spectrally resolved decays and, therefore, found intrinsic size-dependent lifetimes in a Si QD ensemble. The total recombination rate is defined as the inverse of the intrinsic lifetime. The lower the total recombination rate is, the less the nonradiative recombination is involved in the electron–hole recombination.

With known recombination rates, one can evaluate the internal quantum efficiency by comparing with a 100% IQE reference sample. In general, the ensemble PLQY, also referred to as the external quantum efficiency, characterizes the overall optical quality of the sample. It includes contributions from “bright” QDs (100% efficient), “dark” QDs (nonemitting), and “grey” QDs (not 100% efficient in the emitting state). Unlike PLQY, the IQE characterizes the efficiency of only “bright” and “grey” QDs. If “grey” QDs dominate the ensemble, the IQE is less than unity, and the nonradiative processes will limit the resulting PLQY in such QDs.

The obtained curves of the total recombination rates as a function of emission energies are plotted for each sample in Figure 5a. The total recombination rate has a clear dependence of the emission energy for all the curves, following the quantum confinement model. The values in the range near the peak emission energy (∼1.45 eV) of QDs-HSQ and QDs-TES in toluene coincide well with the reference sample of a near-unity IQE,^51^ indicating that there are almost no “grey” QDs in these samples. In contrast, the IQE of QDs-SiO deviates from the reference curve, suggesting the presence of additional nonradiative decay pathways.

**Figure 5:**
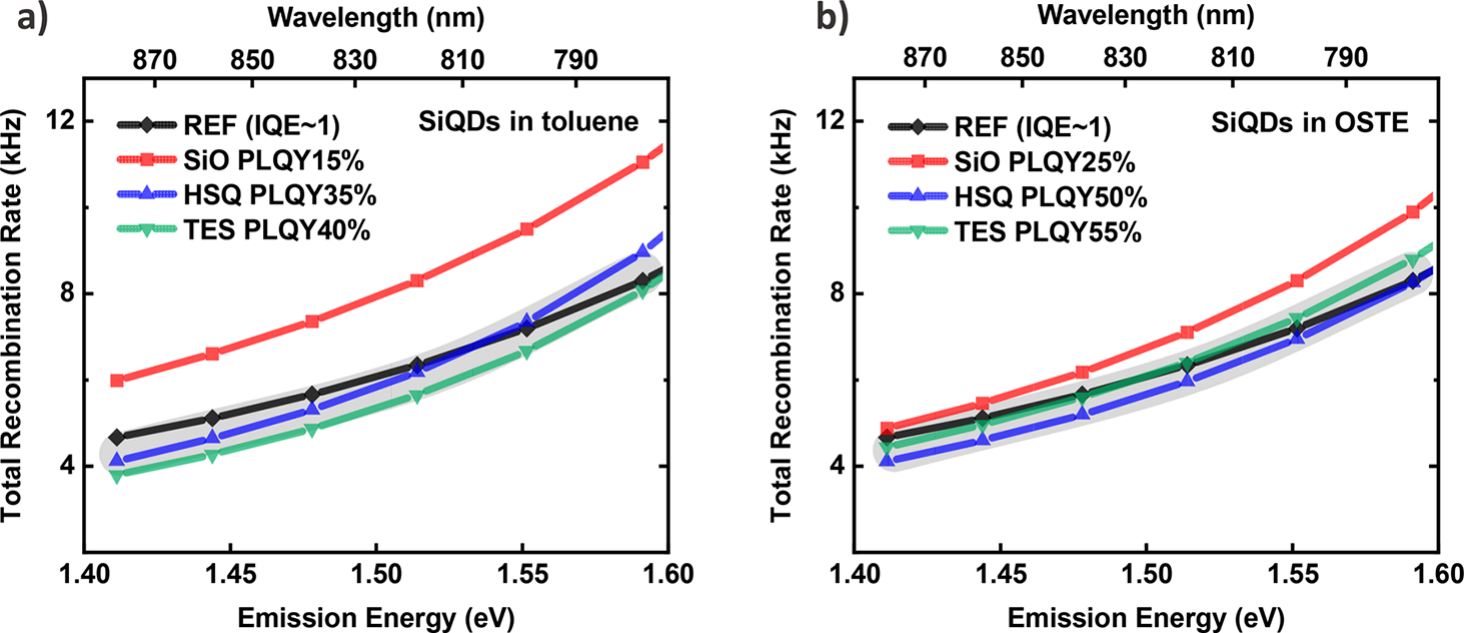
Total recombination rates of Si QDs synthesized from HSQ, SiO, and TES in toluene solution (a) and OSTE matrix (b). The error bar is inside the dot. Note that the reference sample was claimed to have a near-unity internal quantum efficiency in a previous paper. The gray bands at the background indicate that curves included inside have similar values and trends within experimental and analysis errors, revealing their near-unity IQE.

As shown in Figure 5b, the IQEs of QDs-HSQ and QDs-TES were maintained to be near-unity also after the transition from toluene to the OSTE matrix. Therefore, their PLQY enhancements can only originate from partial conversion of “dark” QDs into “bright” ones, probably by further passivation of dangling bonds in the presence of OSTE.^11,48^ For QDs-SiO, the IQE in OSTE still deviates from unity, suggesting persistent nonradiative centers.

To shed light on the origin of these nonradiative processes, we performed attenuated total reflection (FTIR-ATR) measurements on thin dried films of Si QDs. The results are shown in Figure 6a, providing information on the surface properties of these QDs. As can be seen, all of the QDs share the same characteristic peaks. The band at ∼2100 cm^–1^ corresponds to the Si–H stretching mode, and the absorption feature at ∼1100 cm^–1^ can be assigned to the Si–O–Si stretching vibration. Note that theoretical modeling has proven that Si–O–Si bonds can only exist on the Si QD surface and not inside the nanocrystal.^54^ Apart from the coverage of the Si–C bonds (peak unresolved in the spectra) by ligand passivation, there are only Si–O–Si and Si–H bonds on the Si QD surface. Here, there is a consistent one-to-one match between C=O and Si–C bonds, considering the chemical structure of ester ligands. The coverage of Si–H bonds is low, according to the spectra, and can be neglected. Therefore, the ratio between Si–O–Si and C=O (∼1750 cm^–1^) peak areas will reflect relative surface coverage of QDs by the oxide. The ratios of the integrated areas of Si–O–Si and C=O bands are shown in Figure 6b. For QDs-HSQ and QDs-TES, the area ratio is lower than for QDs-SiO, revealing a markedly lower percentage of Si–O–Si coverage on the nanoparticle surface.

**Figure 6:**
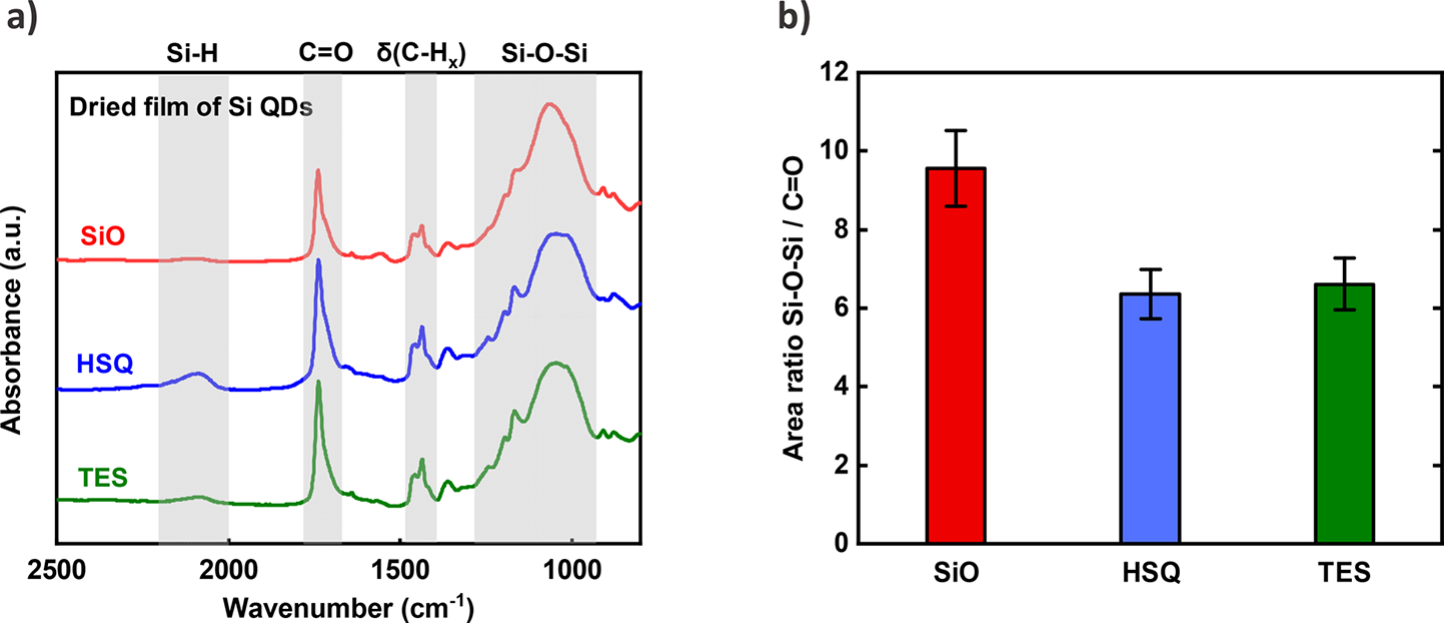
(a) Attenuated total reflectance (ATR) spectra of thin dried films of Si QDs synthesized from SiO (red), HSQ (blue), and TES (green). (b) The ratio of areas under the Si–O–Si and C=O stretching modes obtained from (a).

The oxide on the surface of Si is a known host for charge trap sites, which was well-documented in Si-based nanoelectronics,^55^ and was also explicitly shown for Si QDs in single-dot lifetime measurements.^56^ The IQE and, subsequently, the PLQY are expected to be lower in such a system due to the intermittent trapping and detrapping of carriers, resulting in Auger nonradiative recombinations. The excess of oxide phase and intrinsic nonuniformity of SiO makes it challenging to completely eliminate oxide for such QDs even under prolonged HF etching. As a main fundamental result of this study, we, therefore, conclude that the low PLQY values for SiO-derived QDs stem from the precursor intrinsic structure, limiting this material applicability. On the other hand, TES-derived Si QDs can be controlled through the annealing and etching processes to the quality level matching that of the state-of-the-art precursor HSQ.

In summary, this work introduces a recipe for the synthesis of good-quality, near-infrared emitting Si QDs with ∼55% PLQY for QDs/OSTE nanocomposites (∼40% ensemble PLQY in toluene) and near-unity IQE by using commercial TES as the precursor. By comparing Si QDs of a similar size (4–5 nm) synthesized from HSQ and SiO by structural, chemical, and optical characterizations, we ascribe the superior optical efficiency of QDs-TES to the uniform QD size distribution and a small quantity of oxide on the surface. A relatively well-defined initial structure of TES and the balance of annealing and etching processes developed here made this result possible. Importantly, having a comparable optical quality with their QDs-HSQ counterparts, the cost of QDs-TES is an order of magnitude lower. For applications requiring a large amount of Si QDs, such as large-area semitransparent PVs, QDs-HSQ are prohibitively expensive, while QDs-SiO are prohibitively optically inefficient. Therefore, commercial TES reported here is a promising pathway for the scalable synthesis of high-quality Si QDs. We believe this method will significantly promote applications requiring high loads of Si QDs, especially in a solid phase, thanks to its low cost and good optical efficiency.

## Supporting Information Available

Experimental details; XRD spectra of preannealing powders; low-magnification TEM images of Si QDs; TGA analysis; ATR measurements; synthesis optimization from SiO and TES; reproducibility test; PLQY stability test; PLQY dependency on the excitation wavelength; additional PL decays (PDF)
