# Supplementary material for: Low-Cost Synthesis of Silicon Quantum Dots with Near-Unity Internal Quantum Efficiency
Source: J Phys Chem Lett. 2021 Sep 9;12(37):8909–16. doi: 10.1021/acs.jpclett.1c02187 (PMC8474143; doi:10.1021/acs.jpclett.1c02187)
Supplement: Supplementary file 1 — jz1c02187_si_001.pdf [file jz1c02187_si_001.pdf]

Supporting Information

Low-cost Synthesis of Silicon Quantum Dots with Near-Unity Internal Quantum Efficiency

Jingjian Zhou[1], Jing Huang[1], Huai Chen[2], Archana Samanta[1], Jan Linnros[1], Zhenyu Yang[2,3], Ilya Sychugov[1,*]

[1]Department of Applied Physics, KTH - Royal Institute of Technology, Stockholm 10691, Sweden
[2]MOE Laboratory of Bioinorganic and Synthetic Chemistry, Lehn Institute of Functional Materials, School of Chemistry, Sun Yat-Sen University, Guangzhou 510275, Guangdong, China
[3]Dongguan Institute, Sun Yat-sen University, Dongguan, 523808, China

*Email: ilyas@kth.se

Section S1. Experimental Details

Preparation of TES-derived xerogels

First, in a 100 mL Schlenk flask 10 mL of 90 mmol/L HNO_3 and 10 mL of ethanol were mixed with magneton stirring in an argon atmosphere. Then, 55 mmol (approximately 10 mL) of 95% triethoxysilane (TES) (Sigma-Aldrich, 95%) was slowly added into the flask drop by drop in an ice bath. A transparent gel-like polymer was formed and then aged for 72 hours. Products were purified with ethanol to remove unreacted TES and then transferred to vacuum to remove residual ethanol and water at room temperature for 24 hours. After drying, xerogels were prepared.

Hydrolysis

$$\equiv Si\text{-}O\text{-}C_2H_5 + H_2O \rightarrow \equiv Si\text{-}OH + C_2H_5OH$$

Condensation

$$\equiv Si\text{-}OH + HO\text{-}Si\equiv \rightarrow \equiv Si\text{-}O\text{-}Si\equiv + H_2O$$

Si QDs Synthesis

The synthesis of Si QDs follows a variation of published methods and parameters of annealing differs from one precursor to another.

For TES method, the TES-derived xerogels were annealed at 600°C for 1h to remove residual carbon and solvent and then annealed at 950°C for 1h in a 95% Argon / 5% H_2 atmosphere. To facilitate subsequent etching process, 100 mg of the annealed brown powder together with 3 mL of ethanol was grinded with a mortar and pestle and then transferred into a polypropylene centrifuge tube. 10 mL of 50% aqueous hydrofluoric acid (HF) was then carefully added to the mixture under magnetic stirring. *Caution: HF solution is extremely dangerous. One has to receive professional safety training and specific safety equipment is necessary in operation*. In 1h, the colour turned from dark brown at the beginning to light brown, and no more turbidness can be observed in the solution. Subsequently, 20 mL of toluene was used to extract hydride Si QDs from the aqueous solution for twice (10 mL each), followed by centrifuged at 10000 rpm for 10 min. The precipitate was collected for hydrosilylation.

To obtain ligand-terminated Si QDs, the precipitate was mixed with 2.5 mL of methyl 10-undercenoate (Sigma-Aldrich, 96%), under ultrasonic for 5 min to obtain a homogeneous mixture. The suspension was then transferred to a flask and connected to an argon-filled Schlenk line to prevent from oxygen. The surface passivation reaction was kept at 150°C for 19 h. Si QDs can be extracted from ester solution by adding 4 times volume of antisolvent, which is hexane here, followed by centrifuge at 10000 rpm for 10 min. The precipitate was collected and redispersed in toluene.

For HSQ method, commercial hydrogen silsesquioxane (HSQ) from Applied Quantum Materials Inc. (Canada) was annealed at 1200°C for 1h in a 95% Argon / 5% H_2 atmosphere. 100 mg of the fine Si QDs/SiO_2 powder was mixed with 3 ml of ethanol and 3 ml of DI water subsequently in a polypropylene centrifuge tube under magnetic stirring. A 50% aqueous hydrofluoric acid (HF) solution (3 ml) was then slowly added to the mixture under stirring. The suspension turned yellow after stirring for 1 h, indicating that the Si QDs were released from SiO_2 matrix. The resulted hydride-terminated nanoparticles were collected by extractions with 10 ml of toluene for three times, followed by centrifuged at 10000 rpm for 10 min. The precipitate was then collected for surface passivation. For surface passivation, the fresh hydride

Si QDs were mixed with 3 mL of methyl 10-undecenoate and the mixture was sonicated for 5 min to get homogeneous wetting. Then the suspension was loaded in a flask and transferred to an argon-charged Schlenk line. The reaction mixture was kept at 150°C for 19 h under Ar atmosphere, and the brown suspension changed to a clear orange/brown solution, which was kept directly for further application.

For SiO method, silicon monoxide (SiO) from Sigma-Aldrich was annealed at 920°C for 1h in a 95% Argon / 5% H_2 atmosphere. 25 mg of the fine Si QDs/SiO_2 powder was mixed with 3 ml of ethanol subsequently in a polypropylene centrifuge tube under magnetic stirring. A 50% aqueous hydrofluoric acid (HF) solution (10 ml) was then slowly added to the mixture under stirring. The suspension turned yellow after stirring for 5 h, indicating that the Si QDs were released from SiO_2 matrix. The resulted hydride-terminated nanoparticles were collected by extractions with 10 ml of toluene for three times, followed by centrifuged at 10000 rpm for 10 min. The precipitate was then collected for surface passivation. For surface passivation, the fresh hydride Si QDs were mixed with 2.5 mL of methyl 10-undecenoate and the mixture was sonicated for 5 min to get homogeneous wetting. Then the suspension was loaded in a flask and transferred to an argon-charged Schlenk line. The reaction mixture was kept at 150°C for 19 h under Ar atmosphere, and the brown suspension changed to a clear orange/brown solution, which was kept directly for further application.

Fabrication of Si QDs/OSTE nanocomposite

Pentaerythritol tetrakis (3-mercaptobutylate) was used as thiol monomer and triallyl-1,3,5-triazine-2,4,6 (1H,3H,5H)-trione as allyl monomer. Photo-initiator is 1-Hydroxycyclohexyl phenyl ketone (Irgacure-184). All of them were from Mercene Labs AB, Sweden. First, in a glass vial ~2 mg of Si QDs was dispersed in 0.96 g of allyl monomer under ultrasonication. Then, 3.11 g thiol monomer and 0.04 g photo-initiator were added to the orange solution and mixed thoroughly. The sticky solution was put in an evacuated desiccator to remove the air bubbles trapped inside. At last, the homogeneous solution was cured by 360 nm UV light for 30s to trigger thiol-ene polymerization reaction and stored overnight to complete the polymerization.

Structural and standard absorption measurements

To prepare TEM samples, Si QDs in toluene solutions were drop-casted dropped on a ~15 nm thick amorphous silicon nitride (SiN) support films and dried. Transmission electron microscope (TEM) images of Si QDs were obtained from a JEM-2100F TEM operated at 200 kV. Attenuated total reflection (ATR) spectra were obtained using a Spectrum 100 Fourier transform infrared (FTIR) spectrometer (PerkinElmer, USA) equipped with a Golden Gate diamond ATR (Gaseby Specac Ltd, UK). The spectra were recorded at room temperature with a resolution of 4 cm^{-1} from 4000 to 400 cm^{-1}. Si QDs in toluene solutions were drop-casted dropped on a glass slide and dried until a film of QDs formed. UV-vis absorption spectra were collected on Lambda 750 UV-vis spectrophotometer for Si QDs in toluene solution.

X-ray diffraction (XRD) measurements

XRD patterns were collected with a Rigaku Smart Lab diffractometer (Bragg-Brentano geometry, Cu Kα1 radiation, λ = 1.54056 Å). The spectra were scanned between 2θ ranges of 10–80° with an integration of 350 min.

Thermogravimetric analysis (TGA)

Thermogravimetric analysis (TGA) was conducted on a, Mettler Toledo (TGA/DSC1, Switzerland) instrument. Samples (~10 mg) were placed in alumina pans and heated from 25 to 900 °C with a heating rate of 10 °C min^{-1} in a N_2 flow of 50 mL/min.

Optical measurements

Photoluminescence quantum yield (PLQY)

A home-built integrating sphere setup was utilized to measure absolute PLQY of Si QDs in toluene solution and Si QDs in OSTE matrix. A laser-driven xenon plasma white-light source (Energetic, EQ-99) was used as the excitation source, coupled with a tunable wavelength-selected monochromator (SP2150i, Princeton Instruments). In a 6-inch-diameter integrating sphere (Labsphere), photoluminescence of the excited sample was collected at the bottom port, which was connected through optical fibers to a spectrometer and a Peltier element-cooled CCD camera (-75°C, Princeton Instruments) for signal acquisitions. By using the same monochromatic light source calibrated with an optical power meter (Newport), the system response curve was evaluated for the same spectrometer grating central wavelengths as in measurements. Each spectrum of the sample was calibrated with the system response and then subtracted from the calibrated reference spectrum (without QDs). Through integrations the calculated absorbed and emitted photon numbers per unit time can be obtained. The absolute PLQY is defined as the ratio of absorbed and emitted photon numbers. Another commercial and research-grade QY setups was employed to test the accuracy of this home-built apparatus, as well as benchmarking on organic dyes, such as Rh6G, confirmed the relative error ~ 10%. Unless otherwise specified the excitation at 440 nm was employed in this work.

Micro-photoluminescence (μ-PL) setup and Spectrally Resolved Decay

The excitation beam (a λ_{exc} = 405 nm diode laser) was directed to the sample from outside with a power density of 15W cm^{-2} in a dark-field configuration. The PL emission from the sample was collected by an inverted optical microscope (Zeiss Axio Observer Z1) with a 100x objective lens (Nikon, NA = 0.73 and field-of-view 150 μm) or a 10x objective lens (Zeiss, NA = 0.25 and field-of-view 1.3 mm). It was filtered from the excitation light using a series of 442 and 561 nm long-pass filters (Semrock) and then captured by a spectrometer (Andor Shamrock 500) connected to the left port of the microscope, with a tunable slit at the input and two different gratings of spectral resolution 0.9 and 0.08 nm. A thermoelectrically-cooled EMCCD camera (Andor iXon3 888) was attached to the spectrometer for imaging and an avalanche photodiode (Becker & Hickl, DPC-230) was attached to collect the emitted photons in time-resolved mode. For spectrally resolved decay measurements, a pulsed laser beam was applied with pulse width between 100-500 μs and frequency between 0.3-2 kHz, depending on the selected detection energy. An avalanche photodiode (Becker & Hickl, DPC-230) was used after a spectrometer (Andor Shamrock 500) in order to detect the emitted PL photons collected by a 10x objective lens (NA ≈ 0.25). Through the introduced numerical method in the literature, the spectrally resolved decays of Si NCs were mathematically converted to size-selected decays, excluding the homogenous linewidth broadening in spectrally resolved measurements. Given the E_0 (the ensemble PL center wavelength), ω_{ens} (the width of the ensemble distribution), ω_{hom} (a

homogenous peak width, ~200 meV), and $\tau_{0m}(\varepsilon)$ (the measured lifetime for a certain detection energy ε), by solving an inverse problem the intrinsic lifetime $\tau_{0i}(\varepsilon)$ can be extracted considering the effect of homogenous broadening on the measured lifetime. The total recombination rate is the inverse of the intrinsic lifetime for a certain detection energy. The steps of IQE analysis are listed below:

1. Acquire the PL emission spectrum of the ensemble sample and extract the ensemble PL center wavelength E_0 and the full width at half maximum of the ensemble distribution ω_{ens}. Assume the homogenous peak width ω_{hom} at room temperature is around 200 meV.
2. Measure the spectrally-resolved PL decays of the ensemble sample at a series of detection energies ε at room temperature.
3. Extract the measured lifetime for a certain detection energy $\tau_{0m}(\varepsilon)$ by fitting PL decays with a stretched exponential decay function $I = I_0 \exp\left(-(t/\tau)^{\beta}\right)$, where τ is the lifetime and β is the dispersion factor. The method is applicable for samples exhibiting spectrally resolved decays with a high β values (>0.85).
4. Take out the intrinsic lifetime from the measured lifetime by numerically excluding the homogenous linewidth broadening. Below are main steps:

(1) Assume the initial values of the intrinsic lifetime at four selected detection energies.
(2) Construct four decay curves at selected detection energies using the mathematical function introduced in the REF 51, where the effect of the homogeneous linewidth broadening on the intrinsic lifetime is numerically considered.
(3) Compare the simulated decay parameters with the corresponding measured ones.
(4) Modify the assumed values of the intrinsic lifetime and repeat the above process in several iterations until the simulated parameters coincide well with the measured ones.

5. Plot the obtained intrinsic total recombination rates as a function of the detection energy. The total recombination rate is the inverse of the intrinsic lifetime for a certain detection energy.
6. Compare with a 100% IQE reference sample to establish possible deviations.

Section S2. XRD spectra of pre-annealing powders, low-magnification TEM images, TGA analysis and ATR measurements.

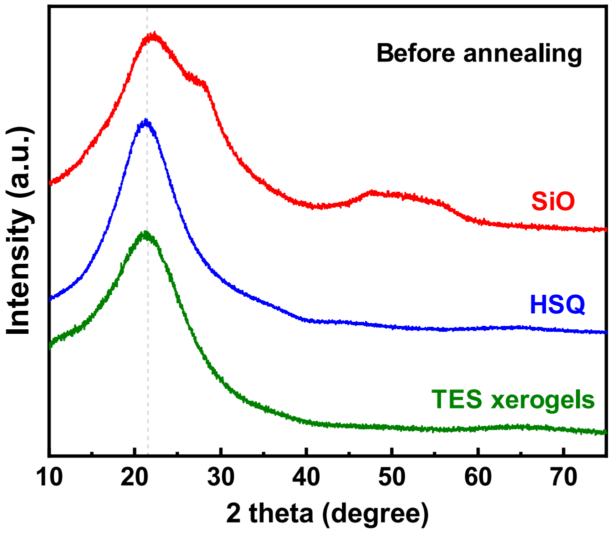

Figure S1. X-ray diffraction (XRD) spectra of three preannealed powders.

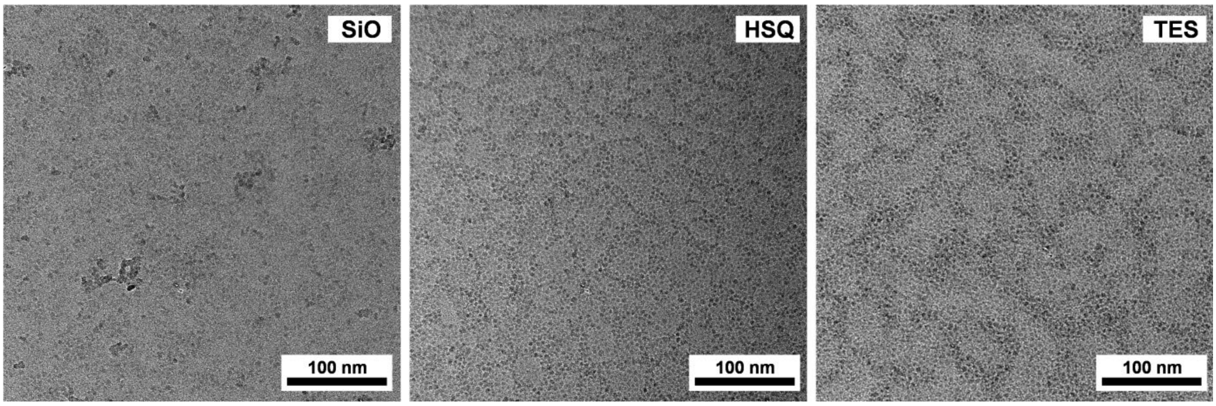

Figure S2. low-magnification TEM images of Si QDs.

The chaos of agglomerations and large particles (＞6 nm) can be clearly observed in the QDs-SiO sample, while not in the other two samples. These particles of poor qualities would lower the ensemble PLQY of the QDs-SiO sample.

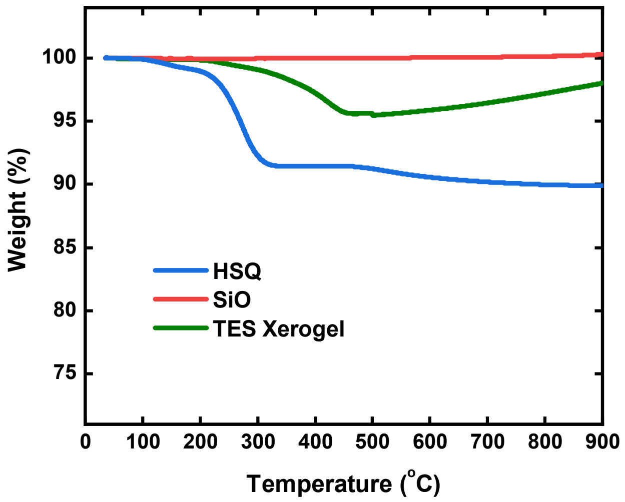

Figure S3. Thermalgravimetric analysis (TGA) curves of HSQ (blue), SiO (red) and TES-derived xerogel (green); heating rate 10°C/min from 30 to 900°C under 100% N_2 atmosphere.

From the TGA curves, SiO barely lose mass during heating from 30 to 900°C under 100% N_2 atmosphere. For HSQ and TES-derived xerogel, they both lose some mass from 130 to 220°C due to moisture, trace solvent and adsorbed CO_2 remained from the beginning. For TES-derived xerogels from 220 to 450°C it is the main loss of heating, which is attributed to the incomplete hydrolysis (residual TES) and incomplete condensation (residual silanol). For the mass loss of HSQ from 220 to 350°C, the cage network distribution and associate loss of SiH_4 are the reason. And for the additional loss from 450 to 700 °C, it can be attributed to the decomposition of SiH_4 to silicon clusters and hydrogen. The latter would be taken away by the flowing atmosphere gas.

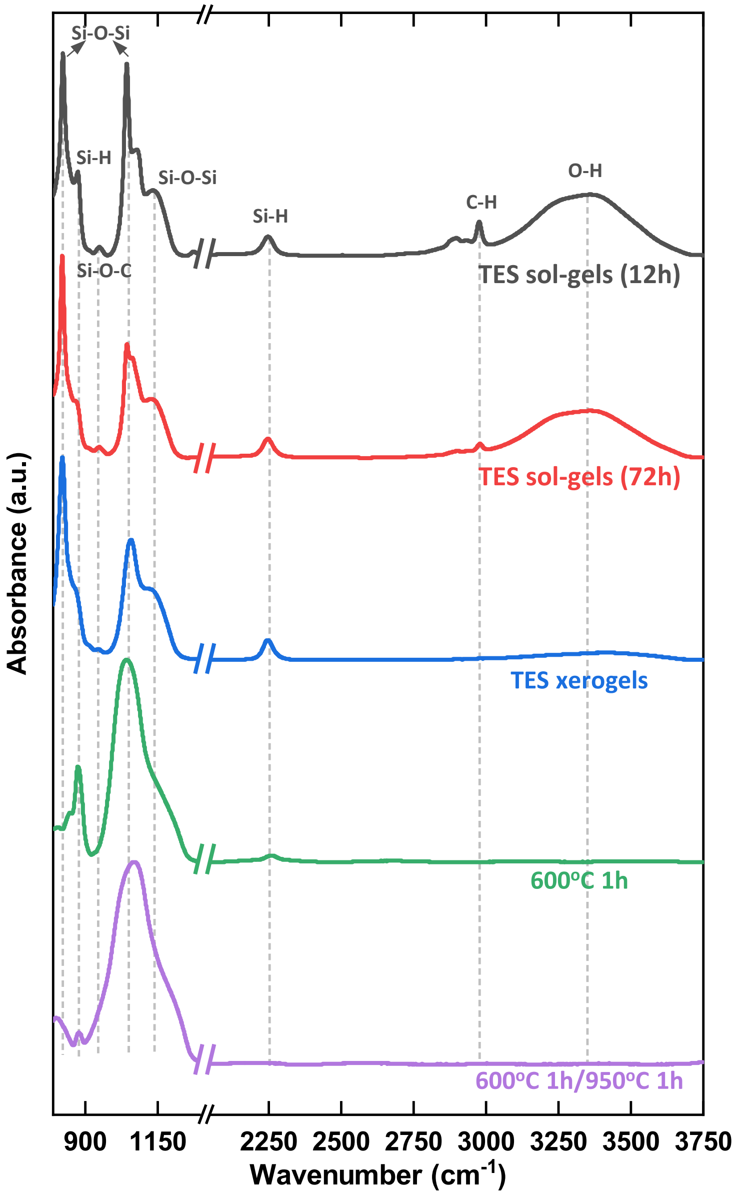

Figure S4. Attenuated total reflectance (ATR) spectra of TES sol-gels after aging for 12h (black), 72h (red), TES xerogels (blue), xerogels annealed at 600°C for 1h (green) and xerogels annealed at 600°C for 1h and 950°C for 1h (purple).

The ATR spectra of TES sol-gels after aging for 12h (black) and 72h (red) were both dominated with ethanol bands, where the broad band at 3400 cm^{-1} (rightmost) refers to the O-H stretching (from incomplete condensation or residual solvent) and the small peak located at 2985 cm^{-1} refers to the C-H stretching. In addition, the Si-O-Si network vibration is responsible for the intense peak at 830 cm^{-1} (leftmost) and the strong band at around 1100 cm^{-1}. The hump at 870 cm^{-1} and the peak at 2250 cm^{-1} both characterize the Si-H bending in the TES sol-gels. At 955 cm^{-1}, the appearance of Si-O-C stretching mode shows that the hydrolysis was incomplete. After drying for 24h under vacuum, the C-H stretching peak vanished from the spectrum (blue) of the TES xerogels (below the detection threshold) while the small hump from Si-O-C still

existed, which either originated from the residual TES or the ethoxy groups bonded to the polymer network (as illustrated in Figure 1). Additionally, the presence of O-H stretching band indicated that the condensation was still incomplete. After annealed at 600°C for 1h, no carbon or ethanol-related bands (below the detection threshold) existed in the spectrum (green), signifying the upgraded main product purity for the subsequent high-temperature annealing. Followed by 950°C annealing for 1h, the Si-H bending band almost disappeared from the spectrum (purple), which was fully consumed as the Si QDs formed. To summarize, the combination of 72h aging, 24h vacuum drying and the preannealing at 600°C for 1h ensured the purity of the polymer product, which should be beneficial to the subsequent formation of Si QDs.

Section S3. Synthesis optimization from SiO

Table S1. Optimizations of QD synthesis from SiO.

Precursor	T	t	Etching time	Amount of HF (mL)	Mass of annealed powder	PL Peak position	PLQY
SiO	1000 °C	20 min	3 h	10 mL	25 mg	840 nm	10±1%
	920 °C	30 min	3 h			850 nm	10±1%
	920 °C	1 h	3 h			870 nm	10±1%
			6 h			820 nm	15±2%

A balance of annealing and etching for synthesis of QDs-SiO was investigated. However, the PLQY of QDs-SiO in toluene was all around 10%, up to 15%. The sample used for comparisons was under conditions of 920°C annealing for 1h and etching for 6 h.

Section S4. synthesis optimization from TES

Table S2. PL peak position as an effect of t_1.

Precursor	T_1	t_1	T_2	t_2	Etching time	PL Peak position
TES	600 °C	0 h	950°C	1 h	0.5 h	960 nm
		1 h				885 nm

Without effective decarbonization, the energy barrier for diffusion of Si atoms would be lower, thereby larger particles being formed.

Table S3. PL peak position as an effect of amount of HF per unit mass of annealed powder.

Precursor	T_1	t_1	T_2	t_2	Etching time	Amount of HF (mL)	Mass of annealed powder	PL Peak position	PLQY
TES	600 °C	1 h	950°C	1 h	1 h	10 mL	50 mg	850 nm	40±4%
							100 mg	850 nm	40±4%
							150 mg	850 nm	40±4%

The relative amount of HF for unit mass of annealed powder has almost no effect on the PL peak position of emission. This implies that the amount of HF used here is far more enough, at least for 150 mg annealed powders.

Table S4. PL peak position as an effect of etching time.

Precursor	T_1	t_1	T_2	t_2	Etching time	Amount of HF (mL)	Mass of annealed powder	PL Peak position	PLQY
TES	600 °C	1 h	1000°C	1 h	1 h	10 mL	50 mg	920 nm	/
					2 h			860 nm	40±4%
					6 h			800 nm	30±3%

The PL peak position is blue-shifted with a longer HF etching time, implying a size reduction of Si QDs.

Table S5. Annealing and etching conditions of green squares in Figure 3.

No.	Annealing	Etching time	PL Peak position	PLQY in Toluene
1	600°C for 1h and 900°C for 1h	0.5h	825 nm	20±2%
2	600°C for 1h and 925°C for 1h	0.5h	850 nm	35±4%
3	600°C for 1h and 950°C for 1h	0.5h	885 nm	35±4%
4	600°C for 1h and 1000°C for 1h	1h	920 nm	/
5	600°C for 1h and 1100°C for 1h	2 h	985 nm	/

Table S6. Annealing and etching conditions of red triangles in Figure 3.

No.	Annealing	Etching time	PL Peak position	PLQY in Toluene
1	600°C for 1h and 950°C for 1h	1h	850 nm	40±4%
2	600°C for 1h and 1000°C for 1h	2h	860 nm	40±4%

Table S7. QD mass yield as an effect of etching time.

No.	Annealing	Etching time	PL Peak center wavelength	PLQY in Toluene	PL emission ratio	Mass yield of etching[a]
1	925°C for 1h	0.5h	~850 nm	~35%	~4.0	~6.5%
2	950°C for 1h	1h	~850 nm	~40%	~2.5	~4%
3	1000°C for 1h	2h	~860 nm	~40%	1	~1.5%

[a]Note that, to calculate mass yields, the relative mass of Si QDs is estimated from PL emission, not from weight. According to the vendor, the mass yield of Si QDs from annealed HSQ is ~10% (this value, of course, can vary slightly from different ligands used for QDs surface passivation), used here as a reference roughly corresponding the PL emission to the mass of Si QDs.

With longer etching time, the mass yield of Si QDs drops. Some Si QDs can be fully etched off with a certain time of etching.

Table S8. PL peak position as an effect of t_2.

Precursor	T_1	t_1	T_2	t_2	Etching time	PL Peak position	PLQY in Toluene
TES	600 °C	1 h	1000°C	1 h	2 h	860 nm	40±4%
				5 h		915 nm	40±10%*

*The measurements error is larger because of the low detector sensitivity above 900 nm.

Prolonging the t_2 would contribute to large size of Si QDs, but not as effective as increasing the annealing temperature. Moreover, the PLQY of Si QDs would not further increase with extended t_2, which implies that 1h annealing at high temperature (T_2) is sufficient enough for forming high-quality Si QDs.

Estimation of QD mass yield (QDs-TES as an example)

From 10mL TES (0.89g/mL), ~2.5 g xerogels can be prepared. The mass loss from annealing is ~8%. So the mass yield from TES to annealed powders is ~25%. As for etching, the mass yield of ething is ~4%. This means ~90 mg Si QDs can be prepared from 2.2 g of annealed powders. In total, the mass yield of QDs-TES from precursor is ~1%.

Section S5. Reproducibilty test, stability test, PLQY dependency on the excitation wavelength and PL decays.

Table S9. Reproducibility of different batches.

Batch	PL Peak Position	PLQY in toluene
one	850 nm	43±4%
second	860 nm	42±4%
third	850 nm	40±4%
fourth	900 nm	32±3%
fifth	855 nm	40±4%
sixth	870 nm	41±4%
seventh	850 nm	39±4%

This QD-TES recipe shows good reproducibility (except the fourth batch with shifted PL peak position).

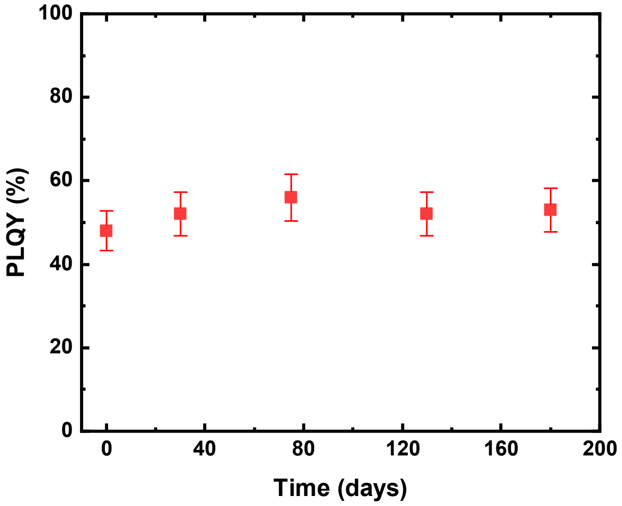

Figure S5. PLQY Stability of Si QDs-TES/OSTE nanocomposite.

The PLQY of QDs-TES/OSTE nanocomposite can be stable for at least 180 days of storage under ambient conditions. The slight variations are attributed to the measurement errors.

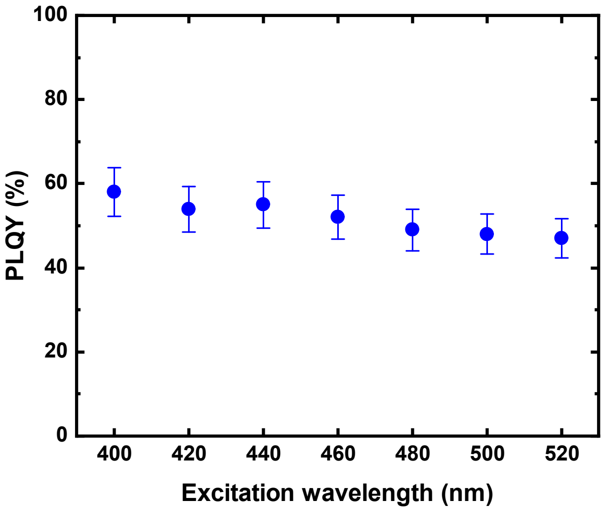

Figure S6. The PLQY dependence on the excitation wavelength of QDs-TES/OSTE nanocomposite.

The PLQY of QDs-TES/OSTE nanocomposite is essentially independent of the excitation wavelength from 400 nm to 520 nm.

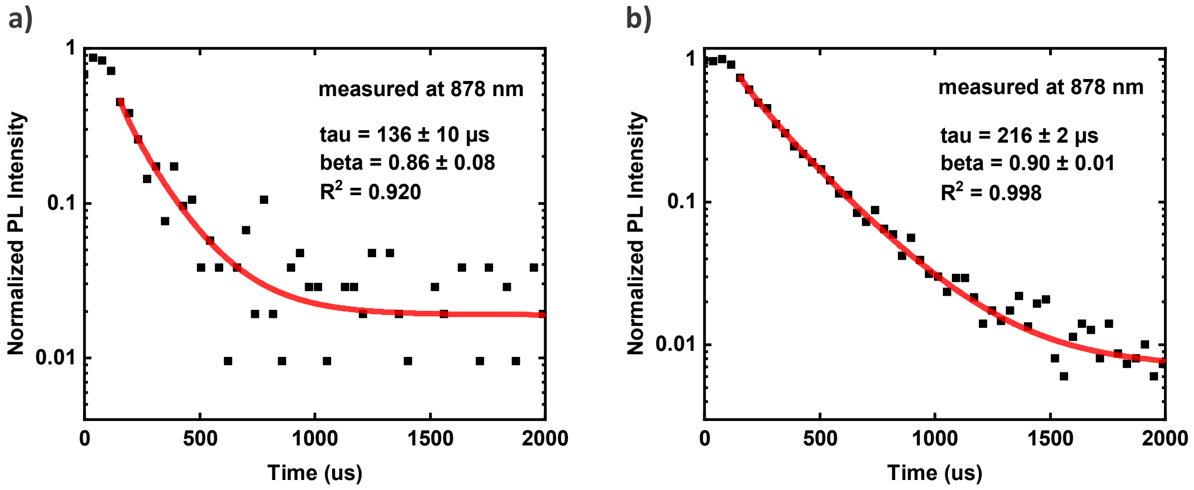

Figure S7. Spectrally resolved PL decays of a) QDs-SiO and b) QDs-HSQ detected at 878 nm and their stretched exponential fits. The signal of QDs-SiO is noisy because of its low quantum yield, corresponding to low PL intensity detected.

Obviously, the PL lifetime of QDs-SiO measured at 878 nm is much shorter than those of QDs-HSQ and QDs-TES and its dispersion factor "beta" is further away from unity.
